# Viral Integration Analysis Reveals Likely Common Clonal Origin of Bilateral HPV16-Positive, p16-Positive Tonsil Tumors

**DOI:** 10.26502/acmcr.96550248

**Published:** 2020-07-13

**Authors:** Lisa M. Pinatti, Heather M. Walline, Thomas E. Carey, Jens Peter Klussmann, Christian U. Huebbers

**Affiliations:** 1 Cancer Biology Program, Program in the Biomedical Sciences, Rackham Graduate School, University of Michigan, Ann Arbor, MI, USA; 2 Department of Otolaryngology/Head and Neck Surgery, University of Michigan, Ann Arbor, MI, USA; 3 Center for Molecular Medicine Cologne (CMMC), University of Cologne, Cologne, Germany; 4 Jean-Uhrmacher-Institute for Otorhinolaryngological Research, University of Cologne, Cologne, Germany

**Keywords:** Viral cancers, HPV, Head and Neck Cancer, Tumor Clonality, Tumor Metastasis

## Abstract

Infections with high-risk human papilloma viruses (HPV) are responsible for a significant number of oropharyngeal squamous cell carcinoma (OPSCC), with infection rates currently rising at epidemic rates in the western world. Synchronous bilateral HPV+ tumors of both tonsils are a very rare event whose understanding, however, could provide important insights into virus-driven tumor development and progression and whether such integration events are of clonal origin. In this study we analyzed a single case of a bilateral tonsillar p16+ HPV+OPSCC. The viral integration status of the various tumor samples was determined by integration-specific PCR methods and sequencing, which identified viral insertion sites and affected host genes. Integration events were further confirmed by transcript analysis. Analysis of the tumors revealed common viral integration events involving the *CD36* gene, as well as a unique event in the *LAMA3* gene which resulted in loss of *LAMA3* exon one in both tissues that had lost the complex viral *LAMA3* integration event. In addition, there were several integration events into intergenic regions. This suggests a common origin but individual evolution of the tumors, supporting the single-clone hypothesis of bilateral tumor development. This hypothesis is further supported by the fact that the two cellular genes *LAMA3* and *CD36* as targets of viral integration are involved in cell migration and ECM-receptor interactions, which provides a possible mechanism for clonal migration from one tonsil to another.

## 1. Introduction

Mucosal human papillomaviruses (HPV) are small double-stranded DNA viruses that infect human mucosa in the oral and anogenital tract. Persistent infection of HPV may lead to a variety of diseases, including genital warts, precancers, and cancers [1]. HPV-associated cancers of the head and neck (HPV+HNSCC), particularly HPV+ oropharyngeal squamous cell carcinoma (HPV+OPSCC), is a growing public health concern; the incidence of this cancer has increased at an epidemic rate in the last few decades [2, 3]. This is in contrast to HPV-negative HNSCC, which is mainly attributed to smoking and has been decreasing in incidence due to public health efforts to decrease smoking rates [2]. As a whole, HNSCC accounts for 650,000 cancer cases and 330,000 deaths annually worldwide [4]. In the United States, three of four newly diagnosed OPSCCs are HPV-positive, and it is predicted that by 2020, the prevalence of HPV+OPSCC will be higher than that of cervical cancer [5, 6]. This increasing incidence is not restricted to the United States but is observed for many countries of the western world including Germany [7].

Persistent oral infection of HPV is a risk factor for the development of OPSCC, but the rates of oral infection in the general population are relatively low and clearance of infections is common [8–10]. It is unclear why some HPV infections are cleared while others persist, but smokers, males, and individuals with higher numbers of sexual partners are more likely to have a persistent oral HPV infection [11]. The tonsils are the most common site of HPV+OPSCC; it is hypothesized that the tonsils act as a reservoir for HPV and the thin epithelium may be more susceptible to infection [9].

HPV drives carcinogenesis through disruption of critical cell cycle regulators TP53 and RB by the viral oncoproteins E6 and E7. Recently, the role of viral integration into the human genome has been investigated as an additional mechanism of carcinogenesis [12]. Viral integration is reported to take place throughout the cellular genome, including both gene-poor and gene-rich regions, and preferentially at fragile sites and regions of microhomology with the viral genome [13, 14]. Integration into or near genes can lead to changes in gene expression through a variety of mechanisms and can also lead to the generation of viral-human fusion transcripts, the implications of which are still widely unclear [12–15].

Very few patients with tonsillar carcinoma present with bilateral HPV+ tumors, and the literature on this phenomenon is somewhat limited [16–20]. A recent retrospective study reported that in a cohort of Danish patients, only 3.3% had synchronous bilateral tonsil cancer [21]. There are multiple hypotheses on the mechanism of this event. One is the single-clone hypothesis, in which carcinoma develops in one tonsil and a clonal population migrates away from the tumor to the other tonsil. Another is that both tonsils develop independent carcinomas due to HPV infection at both sites.

Here we present an interesting case of a 60-year-old Catholic nun who developed synchronous bilateral tonsillar p16+ HPV+OPSCC. Analysis of the tumors revealed both common viral integration events, as well as unique events. This suggests a common origin but individual evolution of the tumors, supporting the single-clone hypothesis of bilateral tumor development. Viral integration occurred into cellular genes *LAMA3* and *CD36*; both of these genes are involved in cell migration and ECM-receptor interactions, which provides a possible mechanism for clonal migration from one tonsil to another.

## 2. Materials and Methods

### 2.1 Tumor Specimens

Formalin-fixed, paraffin-embedded tissue blocks were received as follows: Tumor 1/ block 1B (left tonsil), Tumor 2/blocks 2A, 2B, and 2D (right tonsil). Slides were prepared from each block and the tissues were reviewed by a head and neck pathologist. There was no tumor in block 2B.

### 2.2 DNA/RNA Isolation

10μm sections were taken from FFPE tissue blocks and mounted on a slide. Each section was aligned to the prepared H&E slides to identify the tumor-rich areas, and tissue within the tumor area was microdissected using a scalpel. Following microdissection, DNA and RNA were extracted from the tissue. DNA was isolated using the Machery-Nagel NucleoSpin DNA FFPE kit according to the manufacturer’s protocol. Briefly, paraffin was dissolved with xylene, and the tissue was lysed with lysis buffer and Proteinase K overnight at 56° C. Following overnight digestion, DNA was de-crosslinked, loaded onto the NucleoSpin DNA columns, washed and then eluted in water. DNA concentration was measured using the QUBIT 2.0 Fluorometer.

RNA was extracted from tissue using the Roche High Pure RNA Paraffin Kit according to the manufacturer’s protocol. Briefly, paraffin was dissolved using heptane and methanol, and the tissue was lysed overnight at 56°C with lysis buffer containing Proteinase K and 10% SDS. RNA was extracted using the supplied High Pure Filters and wash buffers, followed by DNase I treatment. RNA was eluted in Elution Buffer and the concentration was measured using the QUBIT 2.0 Fluorometer.

### 2.3 P16 Staining

Tumor tissue sections were stained for p16 using the Roche/Cintec p16 mouse monoclonal antibody (#805–4713).

### 2.4 HPV Testing

HPV types present in the tumors were identified using HPV PCR-MassArray as previously described [22]. In brief, this method detects and identifies 15 high-risk HPV subtypes (16, 18, 31, 33, 35, 39, 45, 51, 52, 56, 58, 59, 66, 68, and 73), 2 low-risk subtypes (6 and 11), and HPV90, considered to be a possible high-risk subtype. The test included interrogation of human GAPDH as a control for sample DNA quality and assay validity. Type-specific, multiplex, competitive PCR was performed to amplify the E6 region of HPV, followed by probe-specific single base extension to discriminate between naturally occurring HPV present in the sample and the synthetic competitors included in the reaction. Matrix-assisted laser desorption/ionization time of flight mass spectroscopy was used for separation of products on a matrix-loaded silicon chip array. Samples were run in quadruplicate with appropriate positive and negative controls.

### 2.5 Detection of Integrated Papillomavirus Sequences (DIPS-PCR)

DIPS-PCR was performed to identify the sites of HPV integration in the genome of the tumors, as previously described [23]. For each tumor, 0.75μg DNA was digested with one of two restriction enzymes, either TaqA1 or Sau3AI. Adapters complementary to the unique overhangs created by restriction digestion were annealed to digested DNA. Linear PCR was performed on each sample using 11 different viral primers to amplify viral fragments. Following linear PCR, exponential PCR using 11 nested viral primers and an adapter-specific primer was performed. All DIPS-PCR primer sequences are listed in Table 2. Products of the exponential PCR reactions were separated by gel electrophoresis (3% agarose gel). Bands were excised from the gel and were purified by Qiagen Qiaquick Gel Extraction Kit. Sanger sequencing of the isolated products was performed by the University of Michigan Sequencing Core, and the results were mapped using NCBI BLAST.

### 2.6 Direct PCR

Primers were designed to amplify the viral/cellular regions of integration identified by DIPS-PCR (Table 3). PCR was performed with genomic DNA from each tumor sample as well as DNA from the DIPS linear PCR reactions in order to enrich for viral products. Amplicons were separated and visualized with gel electrophoresis and were confirmed by Sanger Sequencing of the excised and purified bands.

### 2.7 Transcript Analysis

cDNA was prepared from RNA extracted from the FFPE blocks. cDNA was synthesized from 1μg of RNA using Superscript III and random hexamers. A No-RT control was prepared for each sample to ensure RNA purity. Primers were designed to amplify the native *CD36* and *LAMA3* transcripts proximal to and downstream of each viral integration site using NCBI Primer-BLAST (Table 3). Primers were also designed to amplify across the predicted fusion transcripts (Table 3). RT-PCR was performed using Platinum Taq DNA polymerase. Products of RT-PCR were separated and visualized with gel electrophoresis and were confirmed by Sanger Sequencing of the excised and purified bands.

## 3. Results

### 3.1 Case Report

A 60-year-old female nun in Cologne, Germany presented with bilateral p16-positive OPSCCs of the tonsils. The left tonsil was diagnosed as pT2N2bcM0, the right as pT2N0M0, both Grade 2. Combined radio-chemotherapy was recommended, but only radiotherapy was performed (59.5/50.4Gy) because the patient refused chemotherapy. The patient was free of disease at her last visit to the clinic two years post-diagnosis, after which she was lost to follow up but was reported to have died approximately one year later due to pneumonia.

### 3.2 HPV Testing

**Figure 1:**
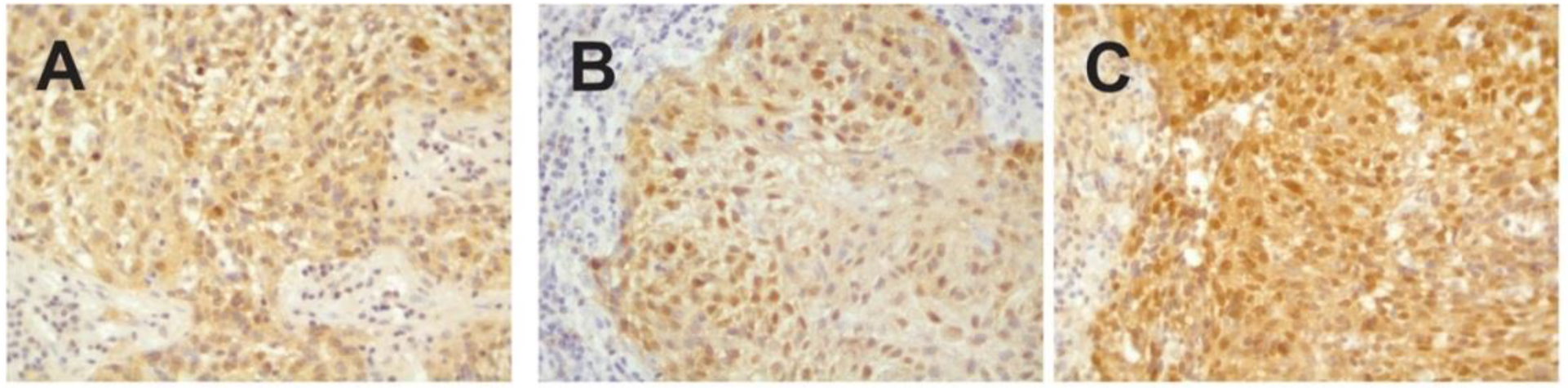
Immunohistochemical staining against p16^INK4a^. A) Left tonsillar tumor (block 1B). B) Right tonsillar tumor (block 2A) and C) Right tonsillar tumor (block 2D). V = 400x.

Staining for p16 was positive for both tumors, and HPV PCR-MassArray determined that HPV16 was the only HPV type present in the tumors (Figure 1).

### 3.3 Integration analysis

**Figure 2:**
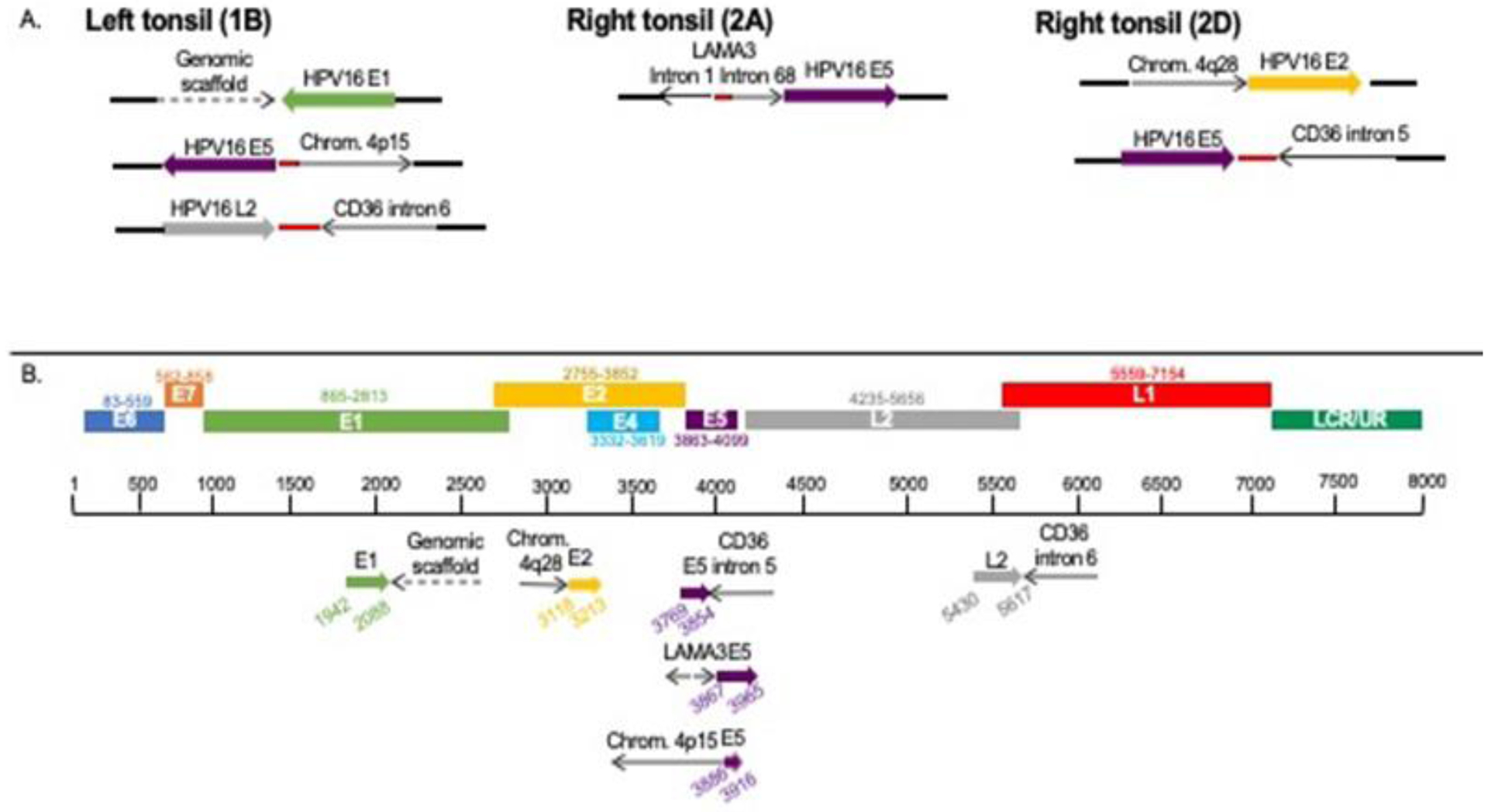
Viral integration sites identified by DIPS-PCR in each tumor block. The viral integration sites of HPV16 into the cellular genome. Red dots indicate unmappable sequence. A. Sites found within each sample. B. Sites from all samples aligned to HPV genome.

DIPS-PCR revealed multiple integration sites in the tumor samples (Table 1). A total of six integration sites were identified; three viral integrations occurred in intergenic or genomic scaffold regions, and three viral integrations occurred in cellular genes (Figure 2A). The tumor from the left tonsil had integration of HPV16 L2 into intron 6 of the cellular gene *CD36*. Two blocks from the right tonsil were analyzed; the tumor cells from the right tonsil had integrations of HPV16 E5 into intron 5 of *CD36*, as well as an additional integration of HPV16 E5 into *LAMA3*, which likely caused a rearrangement of *LAMA3* intron 1 and intron 68. It was curious to note that the segments of integrated HPV could all be aligned linearly starting from E1 to L2 (Figure 2B).

**Figure 3:**
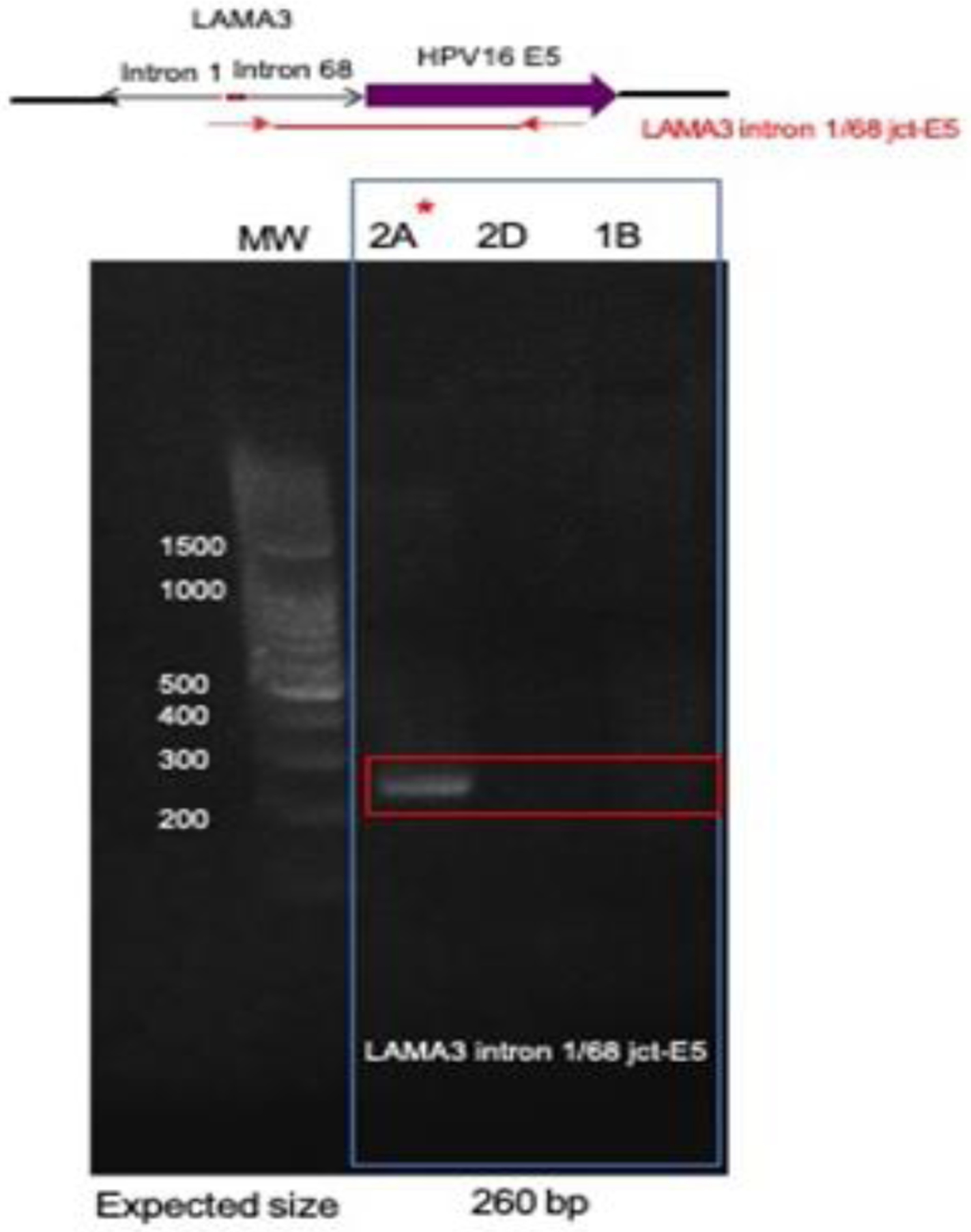
Confirmation of HPV16 integration into LAMA3. Primers designed within the junction of LAMA3 intron 1/intron 68 and HPV16 E5 were used to amplify the identified site in tumor DNA. PCR results, showing the predicted product in only tumor 2A, where the integration was originally identified (indicated by red star). 1B, left tonsil; 2A and 2D right tonsil blocks.

**Figure 4:**
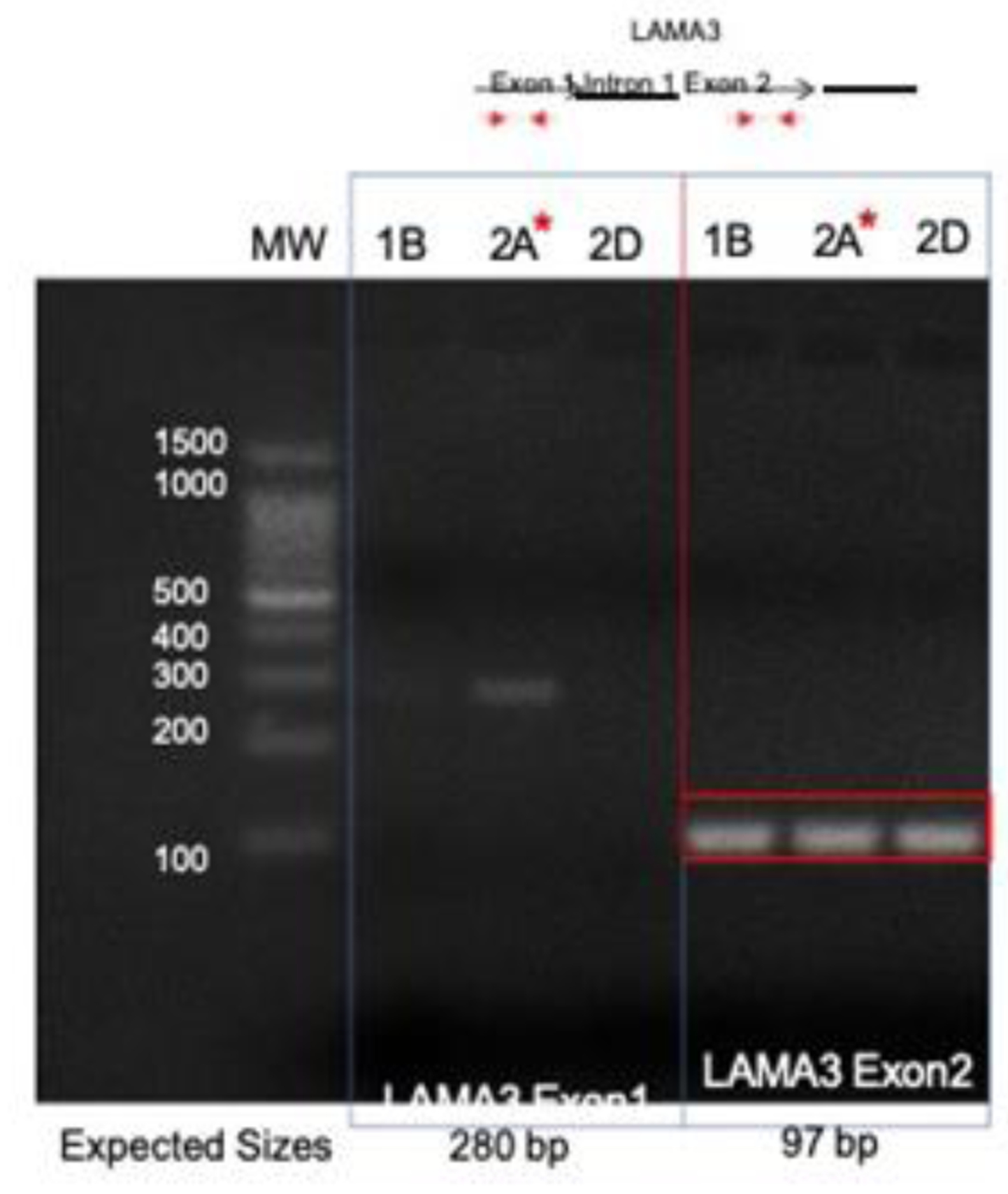
Amplification of native LAMA3 exons in tumor DNA. Primers within LAMA3 exon 1 and exon 2 were used to amplify tumor DNA. A product from exon 1 was present exclusively in tumor 2A (affected by LAMA3 integration), while all three tumors retained exon 2. 1B, left tonsil; 2A and 2D right tonsil blocks.

Direct PCR and Sanger sequencing confirmed the *LAMA3* rearrangement and integration of HPV16 E5 occurred in the right tonsil, but it was not present in the left tonsil or the second block (Block 2D) from the right tonsil, suggesting this rearrangement did not persist in the other intratumoral clonal populations (Figure 3). Amplification of the native exons of *LAMA3* DNA showed that there is an intact copy of *LAMA3* exon 2 present in all 3 samples (Figure 4). However, only tumor 2A, which contained E5 integration into *LAMA3,* showed an intact copy *LAMA3* exon 1.

**Figure 5:**
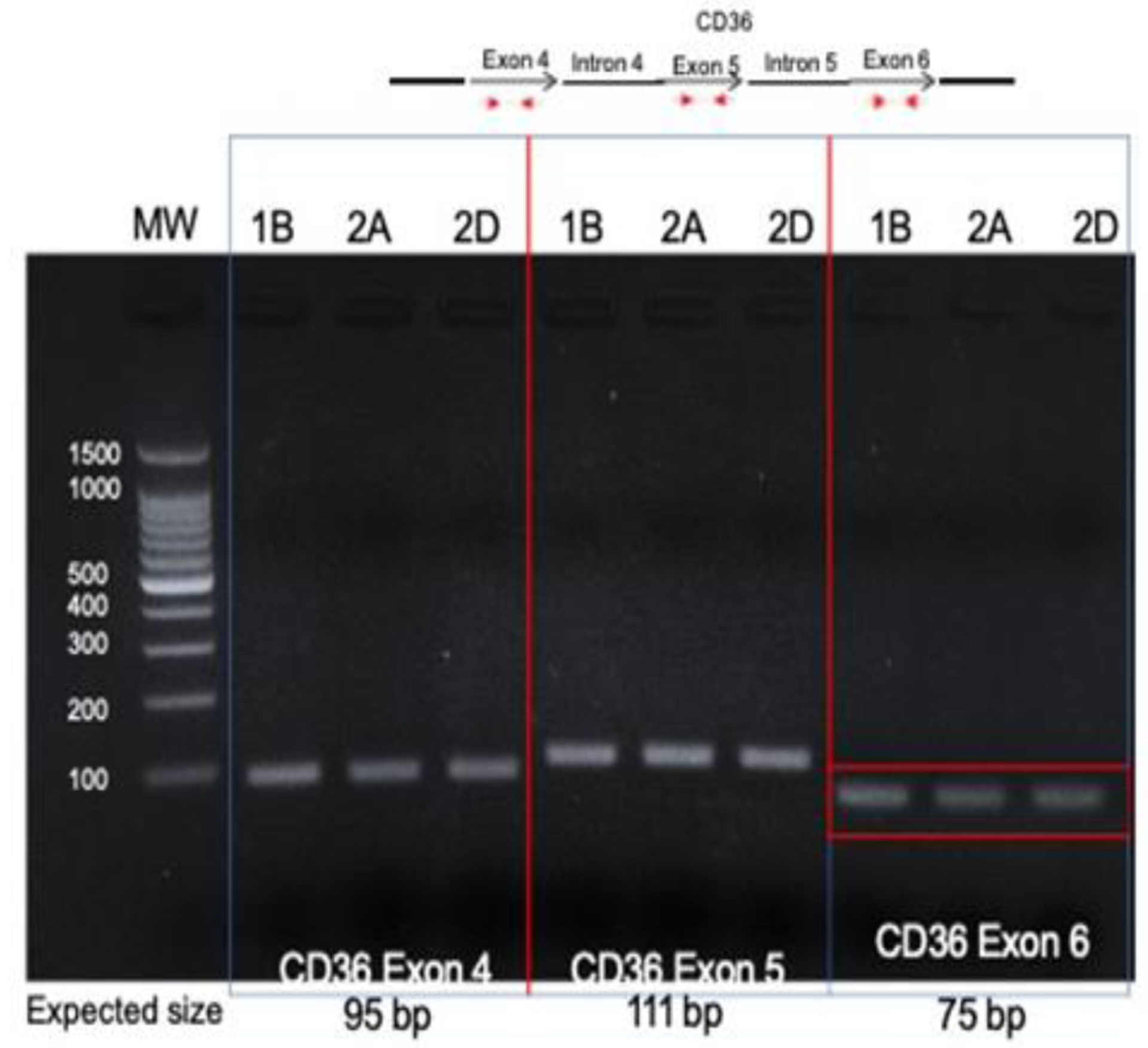
Amplification of native CD36 exons in tumor DNA. Primers within CD36 exon 4, 5 and 6 were designed and used for PCR with tumor DNA from each block. All tumors showed products at the expected sizes for each PCR reaction. 1B, left tonsil; 2A and 2D right tonsil blocks.

Direct PCR of the *CD36* integration site found in the right tonsil (E5-CD36 intron 5) in the other blocks yielded no products; similarly, direct PCR of the site found in the left tonsil (L2-CD36 intron 6) in the right tonsil blocks yielded no products. Amplification of the native exons of *CD36* DNA revealed exons 4, 5, and 6 were present in all blocks (Figure 5).

### 3.4 Transcript analysis

**Figure 6:**
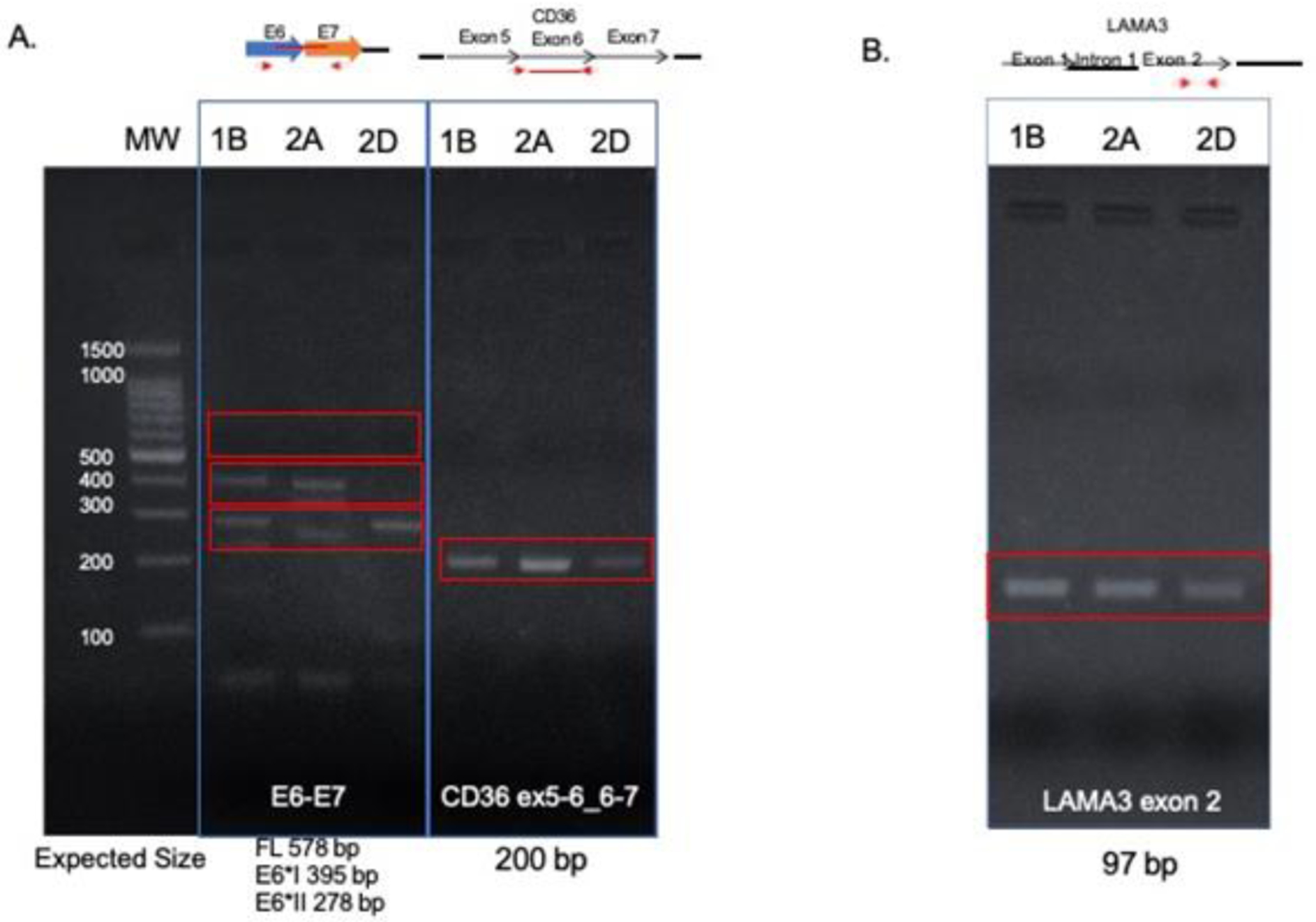
Transcript expression in FFPE tumor tissues. RT-PCR amplification of HPV16 E6-E7 (left) showed E6*I-E7 and E6*II-E7 in blocks 1B and 2A, but only E6*II-E7 in block 2D. RT-PCR amplification of the CD36 exons surrounding the integration sites (right) showed intact copies of CD36 in each sample. 1B, left tonsil; 2A and 2D right tonsil blocks.

HPV16 E6-E7 transcripts were tested and were present in each sample (Figure 6A). Alternate E6-E7 transcripts were visible in each sample, but not full length E6-E7. The left tonsil tumor 1B, and one block from the right tonsil (2A) showed expression of E6*I-E7 and E6*II-E7, but block 2D only expressed E6*II-E7, consistent with evolution of the viral segments with tumor progression.

In order to understand whether viral integration into *LAMA3* and *CD36* disrupted expression of the genes, RT-PCR was performed. RT-PCR revealed that a transcript of the native *LAMA3* exon downstream from the integration site (exon 2) was expressed in all 3 samples (Figure 6B). Normal *CD36* transcripts spanning the two integration sites (the forward primer was designed to amplify the cDNA junction of exon 5–6 and the reverse primer to amplify from the exon 6–7 junction) were also expressed in all 3 samples (Figure 6A). These data suggest there is normal expression of at least one copy *CD36* in each tumor or that the HPV L2 and HPV E5 were spliced out of the transcripts along with introns 5 and 6.

We attempted to amplify viral-host fusion transcripts that may have resulted from viral integration into *LAMA3* or *CD36*. However, RT-PCR using primers designed to amplify the predicted fusion transcripts of *LAMA3* and *CD36* failed to yield any products. Every effort was made to limit the size of the amplicons, as FFPE RNA is usually highly fragmented making it difficult to amplify long products. Therefore, it is unclear whether these fusion transcripts are produced but unidentifiable due to fragmentation, or if they lack a viable promoter and are not expressed.

## 4. Discussion

The incidence of HPV+OPSCC is rapidly rising in the Western world. Despite an overall survival advantage compared to HPV-negative cancers, there is still a significant proportion of patients who develop local or distant recurrences within 5 years and treatment de-escalation has failed [24, 25]. Therefore, there is a critical need to understand the cellular and molecular characteristics of HPV+OPSCCs with unfavorable outcome. Some studies have shown that viral integration of HPV into the genome is associated with worse prognosis [12, 15, 26] and these tumors have a different mutation signature, particularly of *PIK3CA* [27]. Viral integration into cellular genes may lead to disruption of gene expression and generation of viral-human fusion transcripts. Synchronous HPV+ bilateral tonsillar carcinomas are relatively rare with about 40 cases reported in the literature; however, studying their characteristics may contribute to our understanding of OPSCC [18, 21]. There is much controversy in the field whether all HPV+ tonsillar carcinoma patients should have their contralateral tonsil removed in order to prevent missing bilateral disease [28]. Others oppose this idea due to increased morbidity of removing both tonsils and lack of sufficient evidence this would benefit patients, especially given that most patients are given adjuvant radiation and monitored closely so disease in the contralateral tonsil could be caught [29]. The mechanism behind synchronous bilateral carcinoma development is debated; there is evidence for both clonal expansion of a single primary tumor as well as simultaneous development of independent carcinomas due to similar HPV exposure. Understanding how these tumors develop would help inform whether or not all patients with unilateral tonsillar carcinoma are at risk for development of bilateral disease.

Here we have presented a case of synchronous bilateral HPV+ tonsillar OPSCC from a 60-year-old nun. Although the patient claimed to never have sexual contact of any kind, the resulting tumors were HPV+. Viral integration analysis by DIPS-PCR of both tonsil tumors highlighted a number of HPV16 integration sites into both intergenic and genic regions of the cellular genome. There were viral integrations unique to each tonsil as well as within the right tonsil, for which two different areas of the tumor were analyzed. This included the integration of HPV16 E5 into the cellular gene *LAMA3,* which was only present in one block from the right tonsil, suggesting intratumoral heterogeneity.

The *LAMA3* gene containing the viral integration was rearranged and inverted as a result of HPV16 E5 insertion as shown in Figure 2. The implications of the viral integration on *LAMA3* function are unclear because no intact *LAMA3* gene was present in blocks 1B and 2D. Limited analysis of the *LAMA3* gene revealed that there is loss of *LAMA3* exon 1 in the blocks without HPV E5-*LAMA3* integration, but exon 2 is present in all three tumor areas. In trying to understand this observation, we postulated possibilities that might explain these results. If for example, we consider that the initial *LAMA3* integration site was established via a “looping” integration mechanism which reversed the orientation of the *LAMA3* gene and the integrated DNA, including *LAMA3* exon 1, was subsequently excised during clonal expansion, this could explain why block 1B and 2D lost exon 1 DNA, but it is retained in block 2A. This suggests that the *LAMA3* rearrangement included exon 1 and that this was an early event which was later excised as the tumor progressed in the right tonsil (block 2D) and the left tonsil (block 1B), but the only copy of *LAMA3* carried in those tumor cells was the copy missing exon 1.

We also uncovered viral integration into the same cellular gene, *CD36*, in both tonsils. Despite the same cellular gene being involved, different but adjacent regions (E5-L2) of HPV were involved, and the integration sites were located in two different but sequential *CD36* introns (intron 5 and intron 6). Although the locations of integration are slightly different, we expect it would be unlikely to discover viral integration into the same gene in unrelated samples; the majority of samples have unique viral integration sites due to the stochastic nature of viral integration [14, 30]. There is some evidence that viral integration occurs preferentially into genes [31], but it is unlikely that these integrations into *CD36* are the result of separate viral integration events in two independent tumors.

Furthermore, *CD36* is not reported to be a hotspot of HPV integration, in contrast to other genes that have been reported in several cases [14, 32–34]. We believe that these two tumors arose from one primary tumor with viral integration into *CD36* that underwent clonal expansion and was subsequently established in the other tonsil. However, the altered site of integration also suggests the DNA was edited with tumor evolution over time. It is unknown whether viral integration events are stable over time or if they are subject to changes due to either mobile element characteristics or genomic instability. It is also possible that the initial *CD36* integration site was established via a “looping” integration mechanism and some of the integrated DNA was subsequently excised during clonal expansion, as has been described by others [35, 36].

*LAMA3* encodes for the laminin subunit alpha-3, which is one of three members of the complex glycoprotein laminin 5. Laminins are components of the cellular basement membrane, and laminin 5 is reported to be involved in cell adhesion, migration, and the differentiation of keratinocytes [37]. Laminin 5 has also been shown to be overexpressed in invasive oral squamous cell carcinomas but not in premalignant lesions [38, 39]. *CD36*, or cluster of differentiation 36, encodes an integral membrane protein involved in fatty acid import and binds many ligands including collagen, lipoproteins, phospholipids and long-chain fatty acids. Studies done suggest *CD36* may promote cell migration and proliferation in oral cancers and other solid tumors [40–42]. Our analysis suggests there is at least one intact copy of *CD36*, so the functional consequences are unclear. However, both *CD36* and *LAMA3* are involved in ECM-receptor interaction pathways and if their functions were somehow disrupted, this could provide a mechanism for tumor spread. Further investigation of CD36 and LAMA3 protein expression in these samples is warranted to understand whether these proteins are disrupted and whether that may have played a role in metastasis. Our assessment of tumor clonality was limited to HPV genotype and HPV integration site, which other groups have also used to demonstrate tumor clonality [43, 44], but future work will include mutational profiling of cellular genes to strengthen our ability to assess the clonal nature of these samples. This work was somewhat limited by having access to only FFPE tissue, resulting in a lack of quality RNA for integrated viral transcript analysis. This limited our ability to detect gene expression changes of *CD36* and *LAMA3*, as well as detection of the predicted viral-host fusion transcripts. It would have been valuable to evaluate the metastatic lymph node associated with the left pT2N2bcM0 tonsil, but DNA was not available for analysis from that metastatic lesion. Overall, our study supports clonal spread from one tonsil to another, and future work will be focused on validating these results in other bilateral tonsil pairs.

## Figures and Tables

**Table 1:** Integration locations found within the three samples.

Sample	Viral insertion (nt)	Map	Integration locus	Database comparison
Left tonsil (1B)	2088 (E1)	Unplaced	Genomic scaffold	NT_187433.1
	3886 (E5)	4p15	Intergenic	NC_000004.12
	5617 (L2)	7q21	*CD36* intron 6	NG_008192.1
Right tonsil (2A)	3867 (E5)	18q11	*LAMA3* intron 1 - intron 68 fusion	NG_007853.2
Right tonsil (2D)	3213 (E2)	4q28	Intergenic	NC_000004.12
	3854 (E5)	7q21	*CD36* intron 5	NG_008192.1

**Table 2:** DIPS-PCR primer sequences and product size given episomal virus input. Nested PCR reverse adapter primer: 5’-GATGCTGACGACTGATACCGG-3’

Primer ID	1st PCR Primer Name	Primer Sequence	Nested PCR Primer Name	Nested Primer Sequence	Episomal Size
D1	HPV16-E1a	5’-ACGGGATGTAATG GATGGTTTTATG-3’	2nd-HPV16-E1a	5’-AGGGGATGCTATA TCAGATGACGAG-3’	7.5 kb
D2	HPV16-E1b	5’-ATGTTACAGGT AGAAGGGCG-3’	2nd-HPV16-E1b	5’-AGTCAGTATAG TGGTGGAAGTG-3’	7.1 kb
D3	HPV16-E1c	5’-ACGCCAGAATGGA TACAAAGACAAAC-3’	2nd-HPV16-E1c	5’-ATGGTACAATGG GCCTACGATAATG-3’	6.5 kb
D4	HPV16-E2a	5’-ACCCGCATGA ACTTCCCATAC-3’	2nd-HPV16-E2a	5’-TCAACTTGAC CCTCTACCAC-3’	2.7 kb
D5	HPV16-E5a	5’-AGAGGCTGCTGT TATCCACAATAG-3’	2nd-HPV16-E5a	5’-ATGTAGACACA GACAAAAGCAGC-3’	3.0 kb
D6	HPV16-L2a	5’-GTACGCCTAGA GGTTAATGCTGG-3’	2nd-HPV16-L2a	5’-CCAAAAAGTC AGGATCTGGAGC-3’	3.5 kb
D7	HPV16-L1a	5’-ATCCACACCT GCATTTGCTGC-3’	2nd-HPV16-L1a	5’-GCACTAGCATTT TCTGTGTCATCC-3’	5.5 kb
D8	HPV16-E2b	5’-GTGGACATTACAA GACGTTAGCCTTG-3’	2nd-HPV16-E2b	5’-CATGGATATACA GTGGAAGTGCAG-3’	5.4 kb
D9	HPV16-E2c	5’-CGTCTACATGG CATTGGACAGG-3’	2nd-HPV16-E2c	5’-GATAGTGAATG GCAACGTGACC-3’	4.7 kb
D10	HPV16-L2b	5’-CCACTTTACAT GCAGCCTCACC-3’	2nd-HPV16-L2b	5’-CTGTACCCTCTAC ATCTTTATCAGG-3’	3.0 kb
D12	HPV16-E6a	5’-GTATTGCTGTT CTAATGTTGTTCC-3’	2nd-HPV16-E6a	5’-GCAAAGTCATAT ACCTCACGTCG-3’	7.7 kb

**Table 3:** Primer sequences for PCR and/or RT-PCR of viral integration sites and affected genes.

Target	F primer sequence	R primer sequence	PCR Product size (bp)	RT-PCR Product size (bp)
*LAMA3* Exon 1	5’-CATATCCCC GGCTGCGCTA-3’	5’-GCCAGGTTGAA GTAAGTCGGG-3’	280	NA
*LAMA3* Exon 2	5’-CATCCTGTCAC CAATGCCATC-3’	5’-CCAAGGTGAGG TTGACTCTGTT-3’	97	97
*LAMA3* Intron1:68 junction - HPV16 E5	5’-TGTATTTTTAG ATAAAGATGCCGC-3’	5’-ATGTAGACACA GACAAAAGCAGC-3’	260	260
*CD36* Exon 4	5’-TGGGTTAAA ACAGGCACAGAA-3’	5’-ACTTGAATGTTGCT GCTGTTCA-3’	95	NA
*CD36* Exon 5	5’-CGCTGAGGAC AACACAGTCT-3’	5’-GCCACAGCCA GATTGAGAAC-3’	111	NA
*CD36* Exon 6	5’-TGTTCCAAGTCA GAACTTTGAGAG-3’	5’-CAGGGTACGGAA CCAAACTCA-3’	75	NA
*CD36* Exon5–6_6–7	5’-GGCAGCTGC ATCCCATATCT-3’	5’-CCATCTGCAGTA TTGTTGTAAGGA-3’	NA	204
HPV16 L2 - *CD36* intron 6	5’-TGCTGATGCAG GTGACTTTTAT-3’	5’-GCACCTTTCAC AATTTTTAAGGCCA-3’	NA	212
HPV16 E2 - *CD36* intron 5	5’-TACAGTGTCTAC TGGATTTATGTCT-3’	5’-AAACTTACCTCC GTACCAGTA-3’	NA	160
HPV16 E6-E7	5’-GAACTGCAATG TTTCAGGACCCAC-3’	5’-ATTTCATCCTCC TCCTCTGAGCTG-3’	NA	FL 578 E6*I 395 E6*II 278

## Abbreviations:

HPV: human papillomavirus
HPV+OPSCC: HPV-positive oropharyngeal squamous cell carcinoma
HPV+HNSCC: HPV-positive head and neck squamous cell carcinoma
DIPS-PCR: detection of integrated papillomavirus sequences PCR
FFPE: formalin-fixed paraffin-embedded
